# Comparison between posterior dynamic stabilization and posterior lumbar interbody fusion in the treatment of degenerative disc disease: a prospective cohort study

**DOI:** 10.1186/s13018-015-0231-7

**Published:** 2015-06-02

**Authors:** Haodong Fei, Jiang Xu, Shouguo Wang, Yue Xie, Feng Ji, Yongyi Xu

**Affiliations:** Department of Orthopaedics, Huai’an First People’s Hospital, Nanjing Medical University, Huai’an, Jiangsu 223300 People’s Republic of China; Department of Rehabilitation Medicine, Huai’an Second People’s Hospital, Xuzhou Medical College, Huai’an, People’s Republic of China

**Keywords:** Dynamic stabilization, Dynesys, Lumbar spine, Posterior lumbar interbody fusion, Degenerative disc disease

## Abstract

**Background:**

Few studies compared radiographic and clinical outcomes between posterior dynamic stabilization (PDS) and posterior lumbar intervertebral fusion (PLIF) in treating degenerative disc disease (DDD).

**Methods:**

A total of 176 consecutive patients who underwent posterior instrumented spinal surgery for degenerative disc disease between January 2007 and January 2009 were prospectively divided into two groups—PDS and PLIF. All patients included in the analysis were followed up for 3 years. Demographic distribution, perioperative complications, and radiographic and clinical outcomes were compared between the two groups.

**Results:**

The amount of intraoperative blood loss and drained volume was significantly greater in the PLIF group compared with the PDS group (881.1 ml versus 737.4 ml, *p* = 0.004). The length of stay of patients who had PLIF surgery (20.9 days) was significantly longer (*p* = 0.033) than that of patients who underwent PDS surgery (18.9 days). Patients with PLIF surgery had higher total costs than those with PDS surgery (US$12826.8 versus US$11654.5, *p* = 0.002). No statistically significant differences existed in back visual analogue scale (VAS), leg VAS, or Oswestry disability index (ODI) scores between the PDS and PLIF groups of patients at each time point.

**Conclusions:**

Compared with PLIF, PDS have advantages on blood loss, length of stay in hospital, total charges, and radiographic outcomes, but no advantages on leg and back VAS or ODI scores. High-quality randomized controlled trials are still required in the future.

## Introduction

Instrumented fusion in the treatment of degenerative conditions of the lumbar spine is known to have potential complications such as pseudoarthrosis, nonunion, instrumentation failure, infection, donor site pain, and adjacent segment disease [1–3]. The Dynesys Spinal Stabilization System (Zimmer, Inc., Minneapolis, MN, USA) uses pedicle screws, polyethylene-terephthalate cords, and polycarbonate urethane spacers to stabilize a functional spinal unit. The concept of these devices is to reduce the load and restrict the motion of the affected level to stop or delay its degeneration. While studies have indicated positive outcomes with improved Oswestry disability index (ODI) scores and visual analogue scale (VAS) pain scores, as well as shorter recovery time than the fusion for patients with degenerative disc disease (DDD) of the lumbar spine treated with the Dynesys system [4–6], to our knowledge, literature is lacking in studying differences in radiographic and clinical outcomes between posterior dynamic stabilization (PDS) and posterior lumbar interbody fusion (PLIF).

This prospective cohort study was designed to evaluate radiographic and clinical outcomes between posterior dynamic stabilization and posterior lumbar interbody fusion in patients with degenerative disc disease. Furthermore, we hypothesized that PDS would lead to shortened length of hospital stay, reduced medical costs, and fewer perioperative complications.

## Materials and methods

The study protocol was approved by the local institutional review board and ethics committee. Informed consent was obtained from all study participants. A total of 176 consecutive patients undergoing posterior instrumented spinal surgery for degenerative disc disease between January 2007 and January 2009 at a hospital were prospectively divided into two groups according to their clinic sequences. There were 51 males and 44 females subject to PDS, with the mean age of 47.3 years. The implanted level included L2–L5 in 1 case, L3–L4 in 5 cases, L3–L5 in 7 cases, L4–L5 in 45 cases, L4–S1 in 10 cases, and L5–S1 in 27 cases. In the PLIF group, there were 40 males and 41 females, with the mean age of 52.9 years. The implanted level included L2–L5 in 2 cases, L3–L4 in 4 cases, L3–L5 in 5 cases, L4–L5 in 47 cases, L4–S1 in 13 cases, and L5–S1 in 10 cases. The statistical information of patients, including comorbidities, was presented in Table 1. Degenerative lumbar disc disease had been diagnosed in all of these patients, and they all suffered from severe neurogenic claudication and leg, buttock, or groin pain with back pain that was aggravated in sitting or lumbar flexion and relieved by upright position. In all cases, conservative treatment had failed to control these patients’ symptoms. Patients who underwent surgical treatment for nondegenerative conditions (trauma, tumor, or infection) were excluded. We also excluded patients with osteoporosis (bone mineral density T-score less than −2.5) and those who had undergone previous lumbar fusion surgery and those with degenerative deformity.

**Table 1:** Demographics of the groups

Characteristics	Dynesys group (*n* = 95)	PLIF group (*n* = 81)	*p*
Age (years)^a^	47.3 ± 12.9	52.9 ± 11.2	0.002
Sex			0.569
Male	51	40	
Female	44	41	
BMI (kg/m^2^)^a^	26.9 ± 3.9	22.9 ± 3.3	<0.001
Comorbid medical conditions			0.136
Yes	12	17	
No	83	64	
Implanted level, *n* (%)			0.158
L2–L5	1 (1.1)	2 (2.5)	
L3–L4	5 (5.3)	4 (4.9)	
L3–L5	7 (7.4)	5 (6.2)	
L4–L5	45 (47.4)	47 (58.0)	
L4–S1	10 (10.5)	13 (16.0)	
L5–S1	27 (28.4)	10 (12.3)	

Comorbid medical conditions include cardiovascular disorders, respiratory diseases, digestive system disorders, diabetes mellitus, renal insufficiency

*BMI* body mass index

^a^Data are displayed as mean ± SD

On admission to the hospital for the surgery, all patients underwent re-examination including routine lumbar posterior–anterior and flexion–extension position radiographs, lumbar computed tomography (CT), and magnetic resonance imaging (MRI); lumbar myelography was provided for some patients to ascertain whether there was complicated lumbar intervertebral disc protrusion, canal stenosis, or spondylolisthesis. A summary of findings and the clinical history were then documented on a standardized evaluation form and entered into the hospital’s computerized patient records system.

Dynesys implantation was performed in the prone position. A midline approach, with dissection of the erector spinae muscles, provided access to the bony anatomy of the lumbar spine. If indicated, decompression of the spinal canal was performed first. Insertion of the pedicle screws was carried out under radiographic control using a C-arm. The polycarbonate urethane spacer was cut according to the measured distance between the screws, with the length being chosen to compensate for any existing lordosis or kyphosis. The central cord and the spacer were then locked within the screw heads. A soft brace was administered after surgery until wound healing had occurred.

PLIF was performed in a standard manner. Where required, extensive decompression and facetectomy were performed for cage insertion. Autologous bone graft using decompressed bone chips was used within and around the cage, followed by pedicle screw instrumentation and fixation. A posterolateral fusion was also performed for these patients. Surgery was performed by a single senior surgeon. Blood loss was estimated through the evaluation of the amount of blood in the suction canister and that in soaked lap pads. The surgery was performed with the patient under hypotensive anesthesia in which systolic blood pressure was kept lower than 90 mm Hg. All wounds were closed over hemovac drains which were placed on continuous suction. Drains were discontinued no sooner than postoperative day 3, with the output less than 100 ml over 24 h. The same blood transfusion guidelines were used for all patients. Allogenic blood transfusion was performed if hemoglobin decreased to less than 7.0 mg/dL or if anemic symptoms developed, such as a decrease in blood pressure to less than 100 mmHg systolic, tachycardia greater than 100 beats/min, or a low urine output less than 30 mL/h, even after initial fluid challenge with 500 mL normal saline in patients with a hemoglobin level between 7.0 and 8.0 mg/dL [7, 8].

Information was compiled about perioperative complications occurring within 30 days after operation, including surgical complications (postoperative bleeding, wound breakdown, infection, implant failure, neurologic compromise, etc.) and nonsurgical complications (pulmonary disease, arrhythmia, hemodynamic instability requiring inotropic support, etc.). All patients had at least 3 years of follow-up (3.0–5.5 years) physical and radiographic examinations postoperatively.

Data collected included flexion, extension, neutral radiographs, range of motion (ROM) in sagittal view, anterior and posterior disc heights, ODI score, VAS pain score, preoperative BMD, and total charges. Total charge was defined as hospital charges during index hospitalization alone, not including costs for follow-up care and readmission. Radiographs were measured and analyzed by two experienced spine surgeons. Flexion and extension views were taken with patients in the lateral position. The ROM in the sagittal (flexion–extension) view was obtained by the following formula: ROM sagittal = angle (extension) − angle (flexion). Disc height was determined on radiographs taken with the patient in the neutral position and was assessed by measurement of lines drawn at the most prominent points of the endplate anteriorly and posteriorly. Total disc height measured from L1 to S1 was the sum of the disc height at each level. VAS scores were determined on a scale ranging from 0 (no pain) to 100 (worst pain imaginable).

In the PLIF group, fusion status was recorded for each surgically treated segment at each follow-up interval using X-ray film. The level was regarded as fused if there was radiographic evidence of bone bridging the disc space with no lucency. For patients undergoing multiple-level fusion, all surgically treated segments needed to be fused for the patient to be considered as a fusion success.

The data analysis was conducted using SPSS version 19.0 (Chicago, IL, USA). Normal distribution was validated by Shapiro–Wilk test. Continuous data were compared between the PDS and PLIF groups through Student’s *t* test, whereas discontinuous data were analyzed through the chi-squared test. Fisher’s exact test was conducted for small data subsets (*n* < 5). All significance tests were two-tailed, with *p* < 0.05 representing statistical significance. Post hoc power analyses were conducted for the change in VAS, ODI scores, ROM, and anterior disc height and posterior disc height in the PDS and PLIF groups.

## Results

### Surgical characteristics

All variables were in normal distribution. There were no significant differences in mean operation time, mean perioperative red blood cell transfusion unit, and perioperative complications between the two groups (Table 2). However, the mean amount of intraoperative blood loss and drained volume was significantly greater in the PLIF group compared with the PDS group (881.1 ml versus 737.4 ml, *p* = 0.004); in addition, patients who had PLIF surgery had a significantly longer mean length of stay (20.9 days) than patients who underwent PDS surgery (18.9 days) (*p* = 0.033). Mean total charges of patients with PLIF surgery were higher than that of patients with PDS surgery (US$12,826.8 versus US$11,654.5, *p* = 0.002).

**Table 2:** Comparing variables in patients with posterior dynamic stabilization (PDS) and with posterior lumbar interbody fusion (PLIF)

Characteristics	Dynesys group (*n* = 95)	PLIF group (*n* = 81)	*p*
Op time (min)^a^	162.3 ± 41.4	167.3 ± 37.2	0.404
EBL (mL)^a^	737.4 ± 307.2	881.1 ± 373.9	0.004
Transfusion (U/pt)^a^	0.12 ± 0.79	0.09 ± 0.51	0.869
Length of stay (*d*)^a^	18.9 ± 5.3	20.9 ± 6.9	0.033
Total charge (USD)^a^	11654.5 ± 1889.3	12826.8 ± 2946.8	0.002
Complication, *n* (%)	2 (2.1)	4 (4.9)	0.416

*Op time* operation time, *EBL* estimated blood loss, U blood units: *U/pt* units per patient

^a^Data are displayed as mean ± SD

### Clinical outcomes

In the PDS group, the mean VAS scores for back pain obtained preoperatively 1 year after surgery and in the final follow-up visit were 43.7 ± 14.5, 19.5 ± 11.7, and 10.6 ± 9.1, respectively. The corresponding mean VAS scores for leg pain were 51.6 ± 15.7, 13.2 ± 8.9, and 6.7 ± 8.3. The corresponding ODI scores were 57.1 ± 7.7, 33.5 ± 6.6, and 25.9 ± 5.7. In the PLIF group, the mean VAS scores for back pain obtained preoperatively 1 year after surgery and in the final follow-up visit were 45.3 ± 13.9, 18.6 ± 11.3, and 11.4 ± 8.5, respectively. The corresponding mean VAS scores for leg pain were 52.3 ± 16.3, 11.5 ± 8.2, and 7.1 ± 7.5. The corresponding ODI scores were 56.1 ± 8.1, 33.9 ± 7.1, and 24.9 ± 5.9. In both groups, VAS and ODI scores decreased after surgery, and there was a statistically significant difference between preoperative and final follow-up scores for back VAS, leg VAS, and ODI scores (*p* < 0.001). However, no statistically significant differences can be seen in back VAS, leg VAS, or ODI scores between the PDS and PLIF groups of patients at each time point (Table 3).

**Table 3:** Clinical outcome

	Dynesys group (*n* = 95)	PLIF group (*n* = 81)	*p*
ODI (%)^a^			
Pre op	57.1 ± 7.7	56.1 ± 8.1	0.391
Mid-term	33.5 ± 6.6	33.9 ± 7.1	0.738
Final	25.9 ± 5.7	24.9 ± 5.9	0.271
VAS_back_ (mm)^a^			
Pre op	43.7 ± 14.5	45.3 ± 13.9	0.451
Mid-term	19.5 ± 11.7	18.6 ± 11.3	0.633
Final	10.6 ± 9.1	11.4 ± 8.5	0.568
VAS_leg_ (mm)^a^			
Pre op	51.6 ± 15.7	52.3 ± 16.3	0.752
Mid-term	13.2 ± 8.9	11.5 ± 8.2	0.199
Final	6.7 ± 8.3	7.1 ± 7.5	0.782

*VAS* visual analogue scale, *ODI* Oswestry disability index, *VASback* VAS score for back pain, *VASleg* VAS score for leg

^a^Data are displayed as mean ± SD

### Radiological outcomes

#### Range of motion

ROM values of the implanted segment and L1–S1 levels were measured preoperatively, 1 year after surgery and in the final follow-up visit. There were no significant differences in the mean ROM values for the implanted segments and the mean ROM values for the L1–S1 levels between patients in the PDS group and PLIF group preoperatively (6.8° ± 2.7° versus 6.6° ± 3.0°, *p* = 0.579; 18.1° ± 5.9° versus 17.9° ± 6.4°, *p* = 0.852) (Table 4). However, there were statistical differences in the mean ROM values of the implanted segment and L1–S1 levels between the PDS group and the control group (3.5° ± 1.2° versus 0.9° ± 0.9°, *p* < 0.001; 15.3° ± 4.9° versus 13.1° ± 3.3°, *p* = 0.001) (Table 5) 1 year after surgery. In the final follow-up visit, the mean ROM values of the implanted segment and L1–S1 levels in the PDS group were significantly higher than that in the control group (3.7° ± 1.4° versus 0.6° ± 0.6°, *p* < 0.001; 15.8° ± 4.7° versus 13.6° ± 3.4°, *p* < 0.001) (Table 6).

**Table 4:** Preoperative radiographic outcome

	Dynesys group (*n* = 95)	PLIF group (*n* = 81)	*p*
ROM (°)^a^			
Operated level	6.8 ± 2.7	6.6 ± 3.0	0.579
L1–S1	18.1 ± 5.9	17.9 ± 6.4	0.852
Anterior disc height (mm)^a^			
Operated level	12.3 ± 2.4	12.3 ± 2.1	0.796
L1–S1	56.6 ± 4.9	56.7 ± 4.9	0.798
Posterior disc height (mm)^a^			
Operated level	7.7 ± 1.8	7.8 ± 1.2	0.774
L1–S1	36.3 ± 7.2	36.2 ± 4.5	0.911

Disc height was determined on radiographs taken with the patient in the neutral position and was assessed by measurement of lines drawn at the most prominent points of the endplates anteriorly or posteriorly. Total disc height measured from L1 to S1 was the sum of the disc height of each level

*ROM* range of motion

^a^Data are displayed as mean ± SD

**Table 5:** Radiographic outcome at midterm follow-up (1 year postoperatively)

	Dynesys group (*n* = 95)	PLIF group (*n* = 81)	*p*
ROM (°)^a^			
Operated level	3.5 ± 1.2	0.9 ± 0.9	<0.001
L1–S1	15.3 ± 4.9	13.1 ± 3.3	0.001
Anterior disc height (mm)^a^			
Operated level	13.1 ± 2.1	11.9 ± 2.1	<0.001
1 L1–S1	57.1 ± 7.4	56.6 ± 4.8	0.670
Posterior disc height (mm)^a^			
Operated level	8.8 ± 1.5	7.8 ± 1.4	<0.001
L1–S1	38.0 ± 7.0	36.7 ± 5.6	0.171

Disc height was determined on radiographs taken with the patient in the neutral position and was assessed by measurement of lines drawn at the most prominent points of the endplates anteriorly or posteriorly. Total disc height measured from L1 to S1 was the sum of the disc height of each level

*ROM* range of motion

^a^Data are displayed as mean ± SD

**Table 6:** Radiographic outcome at final follow-up

	Dynesys group (*n* = 95)	PLIF group (*n* = 81)	*p*
ROM (°)^a^			
Operated level	3.7 ± 1.4	0.6 ± 0.6	<0.001
L1–S1	15.8 ± 4.7	13.6 ± 3.4	<0.001
Anterior disc height (mm)^a^			
Operated level	12.8 ± 2.2	11.7 ± 2.2	0.001
L1–S1	57.0 ± 4.9	56.2 ± 4.9	0.267
Posterior disc height (mm)^a^			
Operated level	8.3 ± 1.6	7.5 ± 1.3	<0.001
L1–S1	37.9 ± 6.5	37.0 ± 4.5	0.281

Disc height was determined on radiographs taken with the patient in the neutral position and was assessed by measurement of lines drawn at the most prominent points of the endplates anteriorly or posteriorly. Total disc height measured from L1 to S1 was the sum of the disc height of each level

*ROM* range of motion

^a^Data are displayed as mean ± SD

#### Posterior disc height

Posterior disc height was assessed with standing neutral lateral radiographs preoperatively, 1 year after surgery and in the final follow-up visit for both groups. The mean posterior disc height of the implanted segment for the PDS group was 7.7 ± 1.8 mm preoperatively, 8.8 ± 1.5 mm 1 year after surgery, and 8.3 ± 1.6 mm in the final follow-up visit. In the control group, the mean posterior disc height of the implanted segment was 7.8 ± 1.2 mm preoperatively, 7.8 ± 1.4 mm 1 year after surgery, and 7.5 ± 1.3 mm in the final follow-up visit. The mean posterior disc height of the implanted segment was greater 1 year after Dynesys placement than the preoperative one but then gradually decreased; no statistically significant difference was found between the two groups in the preoperative stage and midterm and final follow-up visit in posterior disc heights of the L1–S1 levels, but there was a statistically significant difference between the two groups in the disc height of the implanted segment 1 year after surgery and in the final follow-up visit (*p* < 0.001) (Tables 4, 5, and 6).

### Overall complication rates

In the PDS group, perioperative complications occurred in two (2.1 %) cases, deep venous thrombosis in one case and increasing motor deficit required reoperation in one case. In the PLIF group, perioperative complications occurred in four (4.9 %) cases. Two deaths occurred intraoperatively, one of which was caused by excessive bleeding and the other one of which was caused by massive myocardial infarction. Two broken screws were found (in two patients) and all of the broken screws have been replaced by reoperation. There was no statistically significant difference between two groups (Table 2).

## Discussion

Fusion is generally considered to be the treatment of choice for painful degenerative conditions of the lumbar spine that have proven unresponsive to nonoperative therapy [9, 10]. For a long time, good results were thought to be dependent on radiologically confirmed solid fusion. However, several studies in which patients had pseudarthrosis showing the same clinical outcome as patients with solid fusion have challenged this notion [11–13]. Therefore, it is hypothesized that it is the reduction in (rather than the elimination of) segmental motion brought about by partial fusion or perhaps even simply by an alteration of the structure of spinal tissues or by the surgery itself that results in the alleviation of pain. As pain relief represents one of the most important outcomes in achieving a good result after surgery for degenerative spinal disorders, it would thus appear that solid fusion no longer represents a prerequisite for achieving this goal. If this were so, it would present several advantages, including a reduction in the extensiveness of surgery required and the number of complications, as well as the elimination of undesirable late side effects of fusion, such as adjacent segment degeneration [14] and consequent hypermobility [15].

In our study, back and leg pain VAS scores in the PDS group improved from 43.7 to 10.6 and from 51.6 to 6.7, respectively. In the PLIF group, back and leg pain VAS scores decreased from 45.3 to 11.4 and from 52.3 to 7.1, respectively. In the present study, VAS scores for leg and back pain improved in both groups in the final follow-up visit compared with preoperative scores, indicating that both methods were effective for the treatment of degenerative lumbar diseases. As assessed by multiple measures, patients who underwent a PDS performed as well as those who underwent a PLIF. However, this effect is only evident in the result of 3-year follow-up visit. Many studies [16–18] have reported positive results of the Dynesys, while some reports have indicated that results are no better than those obtained with rigid fusion [19, 20]. We contribute VAS improvement to the biomechanics of the Dynesys, which is a load-sharing device that results in less stiffness than PLIF and may provide immediate stabilization of the diseased segment and neutralize the abnormal forces caused by the pathological bony and soft tissue changes [21–23].

There are conflicting reports on the maintenance of disc height with Dynesys. In our study, changes in posterior disc heights in implant segments showed a contrary pattern of changes compared to those 1 year after surgery and in the final follow-up visit, which were similar to preoperative values. Additionally, CT showed that there was an increase in intervertebral foramen cross-sectional areas and heights immediately after Dynesys implantation but that it subsequently decreased in extent. Thus, no significant difference was evident between preoperative and final follow-up values. It could be concluded from this study that the Dynesys increased spinal canal and intervertebral foramen sizes and local kyphosis with increasing posterior disc height in implanted segments immediately after surgery, but that these returned to their preoperative levels during follow-up visit. However, Yu found that Dynesys implantation resulted in an increase of posterior disc height and that posterior disc height in the follow-up visit 3 years after surgery was maintained at both the operated level and L1–S1 [21]. Beastall et al. reported a reduction of anterior disc height without a significant increase in posterior disc height 9 months after surgery in 24 patients treated with Dynesys [24]. Kim et al. compared the results of single- and multiple-level stabilization with Dynesys in patients with degenerative spinal disease and found no decrease of disc height in either group with a mean follow-up visit of 31 ± 14 months [25].

In line with the studies of Yu et al. [22], our study demonstrated that both blood loss and the length of hospital stay were significantly less in the Dynesys group. The reduction of blood loss in the Dynesys group is presumably caused by the insertion of the Dynesys device requiring less bone and soft tissue dissection as compared to PLIF. Likewise, the shorter hospital stay of patients in the Dynesys group is most likely due to the insertion of the device which is less invasive as compared to PLIF [6, 26]. The latter benefits may be of particular importance for elderly patients, or those with significant co-morbidities.

The present study shows the hospital charge in one institution for PDS and PLIF procedures in hospitalization. The results show a significantly higher cost of patients subject to surgical treatment via PLIF fusion than those with PDS. The mean costs of these two procedures differed by US$1172. The underlying meaning of these results can be interpreted in various ways. First, as the mean hospital cost for both PDS and PLIF surpasses US$10,000, a discrepancy of more than US$1000 in the cost calls into question the real relevance of these data to the economic impact of the decision to use one procedure over the other. Our analysis also suggests another nonmonetary means of measuring value. Though hospital charges were significantly greater for PLIF procedure, there may be no significant differences in charges for materials or services in the operating room. This suggests that the greatest difference in charges for these two procedures may arise from perioperative care. Several potential risk factors may influence the perioperative care such as aging and comorbid medical conditions.

Our data showed no significant differences in complications between two groups, although the percentage of complications in the PLIF group (4.9 %) was higher than that in the Dynesys group (2.1 %). The relatively small sample size may be the reason. Screw loosening and adjacent level instability in the Dynesys group were not significantly greater than those in the PLIF group. Although short-term clinical studies have reported that 6.5 to 20.0 % of patients available for follow-up visit showed screw loosening [20, 27, 28], screw loosening had no adverse impact on clinical improvement. In our study, only two cases of screw loosening occurred in each group and no revision surgeries were required. The lower rate of screw loosening noted in our study as compared with other previously reported studies might be caused by the fact that patients selected for study were relatively young and had normal bone mineral density. Thus, Dynesys system may be especially effective for younger patients for whom surgical intervention is most needed.

Several potential limitations of this study should be mentioned. First, though the follow-up period in this study was 3 years, a much longer follow-up visit is required to fully understand radiographic and clinical outcomes between PDS and PLIF or determine segmental instability at adjacent levels and long-term outcomes. Moreover, the nature of single-surgeon studies raises the possibility that results may not be externally valid and not reproducible elsewhere. Although there is an advantage, i.e., surgical techniques applied throughout the study were consistent and were not contaminated by multiple surgeons performing surgery with inevitable technique differences, variable implants, and inconsistency in bone grafting techniques with the use of substitutes and osteobiologics. In addition, we also acknowledge limitations caused by our patients’ clinical heterogeneity. The difference in age and BMI between two groups may have influence on the result, which will induce bias in the current cohort study.

In conclusion, in this prospective cohort study by a single surgeon, the short-term and long-term functional outcomes clearly demonstrate that PDS have advantages on blood loss, length of stay in hospital, total charge, and radiographic outcomes, but no advantages on leg and back VAS or ODI scores. This indicates that Dynesys stabilization technique requires less extensive surgery than PLIF but does not result in a superior improvement of the clinical outcome compared with PLIF in the last follow-up visit of a minimum 3-year period.
